# Pilot Evaluation of a Fully Automated Bioinformatics System for Analysis of Methicillin-Resistant Staphylococcus aureus Genomes and Detection of Outbreaks

**DOI:** 10.1128/JCM.00858-19

**Published:** 2019-10-23

**Authors:** Nicholas M. Brown, Beth Blane, Kathy E. Raven, Narender Kumar, Danielle Leek, Eugene Bragin, Paul A. Rhodes, David A. Enoch, Rachel Thaxter, Julian Parkhill, Sharon J. Peacock

**Affiliations:** a Clinical Microbiology and Public Health Laboratory, Cambridge University Hospitals NHS Foundation Trust, Cambridge, United Kingdom; b Department of Medicine, University of Cambridge, Cambridge, United Kingdom; c Next Gen Diagnostics, Mountain View, California, USA; d Next Gen Diagnostics, Hinxton, United Kingdom; e Wellcome Sanger Institute, Hinxton, United Kingdom; Memorial Sloan Kettering Cancer Center

**Keywords:** microbiology, *Staphylococcus aureus*, whole-genome sequencing, bioinformatics

## Abstract

Genomic surveillance that combines bacterial sequencing and epidemiological information will become the gold standard for outbreak detection, but its clinical translation is hampered by the lack of automated interpretation tools. We performed a prospective pilot study to evaluate the analysis of methicillin-resistant Staphylococcus aureus (MRSA) genomes using the Next Gen Diagnostics (NGD) automated bioinformatics system. Seventeen unselected MRSA-positive patients were identified in a clinical microbiology laboratory in England over a period of 2 weeks in 2018, and 1 MRSA isolate per case was sequenced on the Illumina MiniSeq instrument. The NGD system automatically activated after sequencing and processed fastq folders to determine species, multilocus sequence type, the presence of a *mec* gene, antibiotic susceptibility predictions, and genetic relatedness based on mapping to a reference MRSA genome and detection of pairwise core genome single-nucleotide polymorphisms. The NGD system required 90 s per sample to automatically analyze data from each run, the results of which were automatically displayed. The same data were independently analyzed using a research-based approach. There was full concordance between the two analysis methods regarding species (S. aureus), detection of *mecA*, sequence type assignment, and detection of genetic determinants of resistance. Both analysis methods identified two MRSA clusters based on relatedness, one of which contained 3 cases that were involved in an outbreak linked to a clinic and ward associated with diabetic patient care. We conclude that, in this pilot study, the NGD system provided rapid and accurate data that could support infection control practices.

## Abstract (precis)

Genomic surveillance that combines bacterial sequencing and epidemiological information will become the gold standard for outbreak detection, but its clinical translation is hampered by the lack of automated interpretation tools. We performed a prospective pilot study to evaluate the analysis of methicillin-resistant Staphylococcus aureus (MRSA) genomes using the Next Gen Diagnostics (NGD) automated bioinformatics system.

## INTRODUCTION

Detection of outbreaks associated with methicillin-resistant Staphylococcus aureus (MRSA) in health care settings at the earliest opportunity supports early interventions to bring these to a close. Outbreak detection in routine practice relies on the daily identification of patients who carry or are infected with MRSA and collation to determine relatedness in time and place. In the event that 2 or more MRSA-positive patients have epidemiological links, all of the available information is then reviewed to determine the probability of an outbreak and the need for further investigations, including screening of other patients, staff members, and/or equipment, and interventions such as enhanced infection control measures and cleaning. MRSA outbreaks may take several days or weeks to detect and to investigate using this reactive approach.

An alternative paradigm is to undertake routine, prospective, whole-genome sequencing (WGS)-based surveillance of MRSA and to use comparisons of whole genomes to drive infection control outbreak investigations and interventions (1). Following sequencing, the decision to investigate (or not) could be driven by a desk-based analysis of bacterial relatedness and patient movement data (1). There is growing evidence for the potential to transform infection control practices through the incorporation of bacterial sequence data (2–6). MRSA sequencing, when used in combination with patient movement data, provides a more accurate determination of transmission events and outbreak status than standard infection control methods alone (2–4). Furthermore, a study of genomic surveillance of MRSA strains isolated in a large clinical microbiology laboratory in the east of England over 12 months led to the identification of hundreds of transmission clusters that were not detected by standard infection control methods (4).

Large routine microbiology laboratories are already capable of supporting pathogen sequencing, but a major impediment to its clinical translation is the lack of fully automated interpretation tools. Here, we report the findings of a prospective pilot study to evaluate the analysis of MRSA genomes using the Next Gen Diagnostics (NGD) automated bioinformatics system, which undertakes rapid pairwise comparison of genomes to provide a similarity matrix and has the potential to collate this matrix with patient movement data to provide infection control units with rapid outbreak visualization.

## MATERIALS AND METHODS

### Ethical approval.

The study was conducted under ethical approval from the National Research Ethics Service (reference no. 11/EE/0499) and the Cambridge University Hospitals NHS Foundation Trust (CUH) Research and Development Department (reference no. A092428).

### Study setting, patients, and sample identification.

The study was conducted at the Clinical Microbiology and Public Health Laboratory at the CUH in the United Kingdom. MRSA-positive patients with samples submitted over a period of 2 weeks in 2018 were identified using the hospital information technology system (EPIC Hyperspace 2014; Epic Systems Corp.). Putative or confirmed MRSA-positive culture plates were retrieved by the study team and were confirmed to be S. aureus using the Staph Latex kit (Pro-Lab Diagnostics). Laboratory data on the date and place of sampling, the sample type (screen or clinical sample), bacterial identification, and susceptibility test results were collected. Susceptibility testing using the European Committee on Antimicrobial Susceptibility Testing (EUCAST) disc diffusion method is routinely performed in the laboratory (7), and such data were also collected. Epidemiological data on ward location (for inpatients), general practitioner (GP), and residential postal code were collected.

### Whole-genome sequencing.

Patients and samples were renumbered with an anonymous study code, after which each patient had a single anonymized MRSA isolate processed for WGS. For each isolate, a 1-μl loopful of growth was inoculated into phosphate-buffered saline to form a suspension and DNA was extracted using the Qiagen DNA mini extraction kit. Sequencing libraries were prepared using the Illumina Nextera DNA flex kit and sequenced with an Illumina MiniSeq system with a run time of 13 h, using the high-output 150-cycle MiniSeq cartridge and the Generate Fastq workflow. Each run contained 3 controls (no-template control, positive control [MRSA MPROS0386], and negative control [Escherichia coli NCTC12241]).

### Quality control metrics.

Controls were required to pass the following metrics prior to further analysis: MRSA positive control—highest match to S. aureus using Kraken, <1% contamination with another species (equating to <0.4% match in Kraken) (8), assigned to sequence type 22 (ST22), *mecA* detected, minimum mean sequence depth of 20×, and minimum of 80% mapping coverage of the MRSA reference genome (HO 5096 0412); E. coli negative control—highest species match to E. coli in Kraken, *mec* not detected, and no S. aureus ST assigned; no-template control—<1% contamination with any bacterial DNA (equating to >95,000 fragments) (8). MRSA isolates from the test panel were required to pass the following metrics prior to further analysis: highest match to S. aureus using Kraken, <10% contamination with another species (equating to <4% match in Kraken) (8), assigned to the correct ST, *mec* gene detected, minimum sequence depth of 20×, and minimum of 80% mapping coverage of the MRSA reference genome (HO 5096 0412).

### Sequence data analysis using standard bioinformatics pipelines.

Bacterial species were determined using Kraken v1 (https://ccb.jhu.edu/software/kraken) and the miniKraken database (https://ccb.jhu.edu/software/kraken/dl/minikraken_20171019_8GB.tgz). Multilocus STs were identified for MRSA using ARIBA v2.12.1 (https://github.com/sanger-pathogens/ariba/wiki/MLST-calling-with-ARIBA). MRSA strains were screened for the presence of *mecA* (GenBank accession no. HE681097, positions 2790560 to 2792566), *mecB* (GenBank accession no. AP009486, positions 25508 to 27532), or *mecC* (GenBank accession no. FR821779, positions 35681 to 37678) using ARIBA, with a minimum percentage identity of 70% required, based on the report by Ito et al. (9), and a minimum of 90% of the gene length covered. Isolates were mapped to clonal complex (CC)-specific references when there was more than 1 isolate belonging to the same CC (CC22, MRSA HO 5096 0412 [GenBank accession no. HE681097]). Mapping was performed using SMALT (https://www.sanger.ac.uk/science/tools/smalt-0), with mapping and base calling performed as described by Klemm et al. (10) with the following modifications: kmer size, 13; step size, 6. Mobile genetic elements were removed from the alignment using an available file (https://figshare.com/articles/Mobile_genetic_elements_on_the_ST22_strain_HO_5096_0412/7059365) and script (https://github.com/sanger-pathogens/remove_blocks_from_aln). Single-nucleotide polymorphisms (SNPs) were identified using an available script (https://github.com/sanger-pathogens/snp-sites). The depth and percentage coverage of the mapping reference were determined using an available script (https://github.com/sanger-pathogens/vr-codebase/blob/master/modules/VertRes/Pipelines/Mapping.pm). The following parameters were used to identify SNPs: minimum number of reads matching the SNP, 4; minimum number of reads matching the SNP per strand, 2; ratio of SNP base to alternative base, 0.75; variant quality, 50; mapping quality, 30. A database of previously reported (11, 12) resistance-conferring genes and mutations was created, and the sequence reads for each test isolate were screened for their presence or absence. The bioinformatics tool ARIBA v2.12.1 (https://github.com/sanger-pathogens/ariba) was run with default parameters, with sequence reads and the database as input. The outputs of the ARIBA runs for individual isolates were collated to generate a summary presence/absence table.

### Sequence data analysis using an automated system.

The cloud-based NGD bioinformatics system (NGD, Mountain View, CA) automatically self-activated on completion of the 13-h MiniSeq run and uploaded the raw reads to a 64-computer Amazon Web Service cloud system. The 24 fastq files generated during a single run were processed and analyzed within 35 min (∼90 s per file/sample). Automated analyses identified the bacterial species, determined the presence of a *mec* gene, predicted susceptibility, assigned the ST, and determined relatedness by mapping to a reference MRSA genome from which mobile genetic elements had been removed. The raw reads were trimmed with Trimmomatic v.0.36-4, to remove low-quality bases with quality scores of <10 from the ends of each read and to filter out reads with average base pair quality scores of <20. The resulting pool of reads was then mapped to a reference with SMALT v0.7.6. Variants were called with Samtools v1.3.1 and Bcftool v1.3.1, and mobile genetic elements were masked out. Each site was then compared for every pair of isolates, taking into account evidence supporting both reference and alternative alleles, with a proprietary NGD pipeline module. Each isolate was also assessed, using an integrated and curated resistome database, for the presence of predefined genes and mutations that are known to confer resistance. Resistance and susceptibility predictions were automatically generated. Isolates were flagged as passed or failed on the basis of having 20× coverage depth over at least 80% of the mapping reference genome.

### Data availability.

The study sequences were deposited in the European Nucleotide Archive (https://www.ebi.ac.uk/ena), with accession numbers ERS3414305, ERS3414308, ERS3414304, ERS3414296, ERS3414298, ERS3414303, ERS3414300, ERS3414292, ERS3414293, ERS3414294, ERS3414295, ERS3414291, ERS3414306, ERS3414307, ERS3414309, ERS3414297, and ERS3414299 (also see Table S1 in the supplemental material).

## RESULTS

We identified 17 MRSA-positive individuals over a 14-day period in 2018 with isolates available for sequencing, which were cultured from samples submitted to our diagnostic microbiology laboratory from hospital wards and clinics (*n* = 15) and GP surgeries (*n* = 2). Eight samples were multisite MRSA screens (swabs of nose, throat, and groin) and 9 were diagnostic specimens (8 surface swabs and 1 tissue specimen) (Table 1).

**TABLE 1:** ST, specimen type, and genetic relatedness^*a*^

Sample no.	Multilocus ST	Specimen type	Genetic cluster (<50 SNPs different)
HICF0049	22	Multisite screen	2
HICF0056	22	Multisite screen	
HICF0059	22	Multisite screen	1
HICF0060	5	Wound swab	
HICF0062	22	Multisite screen	1
HICF0064	22	Multisite screen	2
HICF0068	22	Multisite screen	
HICF0150	22	Wound swab	
HICF0151	1	Genital swab	
HICF0152	22	Tissue	1
HICF0153	45	Ulcer swab	
HICF0154	22	Multisite screen	1
HICF0155	22	Wound swab	1
HICF0156	22	Ulcer swab	
HICF0157	97	Throat swab	
HICF0158	22	Skin swab	
HICF0159	22	Multisite screen	2

a The results shown were concordant between the NGD tool and the research bioinformatics method.

The research informatics method and the NGD tool were used in parallel to identify bacterial species, to assess the presence of a *mec* gene, to assign the ST, to determine genetic relatedness based on pairwise SNPs, and to predict antibiotic susceptibility. Each analysis was independently performed. There was full concordance between the two analysis methods regarding species (S. aureus), detection of a *mec* gene (*mecA* in all cases), and ST assignment for the 17 isolates (see Table S1 in the supplemental material). Phenotypic drug susceptibilities to 10 antibiotics were compared with genetic resistance prediction by the NGD tool and the research bioinformatics method (Table 2). Both methods were concordant with the phenotypic susceptibility results with the exception of fusidic acid testing for 1 isolate (resistant by phenotype and susceptible by genotype), equating to overall concordance between phenotype and genotype results of 99.3% for both informatics methods. The discrepant isolate was not available for repeat testing.

**TABLE 2:** Comparison between phenotypic susceptibility testing and genetic prediction for 10 antibiotics

Antibiotic	No. of cases						Concordance (%)		
	Phenotype^*a*^		Genotype						
			NGD		Research analysis		NGD vs phenotype	Research analysis vs phenotype	NGD vs research analysis
	R	S	R	S	R	S			
Methicillin	17	0	17	0	17	0	100	100	100
Erythromycin	9	7	9	7	9	7	100	100	100
Fusidic acid	3	13	2	14	2	14	93.75	93.75	100
Gentamicin	0	16	0	16	0	16	100	100	100
Rifampicin	0	16	0	16	0	16	100	100	100
Tetracycline	4	12	4	12	4	12	100	100	100
Chloramphenicol	0	15	0	15	0	15	100	100	100
Ciprofloxacin	12	3	12	3	12	3	100	100	100
Linezolid	0	15	0	15	0	15	100	100	100
Mupirocin	0	15	0	15	0	15	100	100	100
Total	45	97	44	98	44	98	0.99	0.99	100

a R, resistant; S, susceptible.

**FIG 1:**
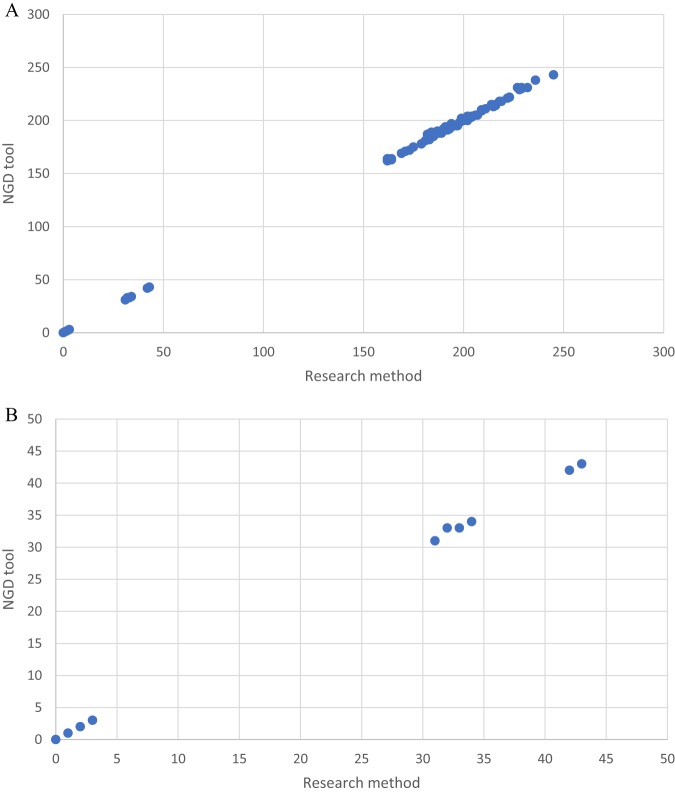
Comparison of SNP differences identified between study isolate pairs using research analysis (*x* axis) and the NGD tool (*y* axis) for ST22 isolates. (A) All comparisons. (B) Comparisons for isolates <50 SNPs apart, based on either method.

Four STs were identified, with ST22 predominating (*n* = 13), together with single representatives of ST1, ST5, ST45, and ST97 (Table 1). A comparison of the pairwise SNP differences for the research informatics pipeline and the NGD tool is shown in Fig. 1. A head-to-head comparison demonstrated that, for ST22, there was a median of 0 SNPs (range, 0 to 5 SNPs) differing between the two methods. SNP results were identical for 33/78 pairs (42%), while the NGD tool identified between 1 and 5 differential SNPs more in 23/78 pairs (29%) and 0 to 2 SNPs fewer in 22/78 pairs (28%), compared to the research pipeline, which was used as the gold standard. Investigation of the sites at which the NGD tool identified fewer differential SNPs than the research pipeline revealed 4 locations that were not qualified for comparison with the NGD tool due to insufficient depth of coverage (*n* = 1; 6×), different mapping quality scores (*n* = 1; 29 with the NGD tool and 35 with the research pipeline), and lack of reads mapping in both directions (*n* = 2). In investigating where the NGD tool identified more differential SNPs, it was found that, for 8 SNP locations across 8 isolates, the NGD tool called the reference base, while the research pipeline called an unknown base, disqualifying the cases from the research pipeline comparison. These differences were predominantly due to the AF1, allele ratio, strand bias, and/or map bias cutoff values, which are not used by the NGD tool. Analysis of these locations revealed that the majority of reads supported the reference allele in the research pipeline, matching the findings of the NGD tool. There were insufficient non-ST22 isolates to support SNP comparisons between the pipelines for other STs.

Initial clustering was performed based on isolates that were ≤50 SNPs different from triage cases for more detailed genomic and epidemiological analysis, as described previously (4). Both analysis pipelines identified two clusters, containing 5 and 3 isolates/patients (Table 1). For cluster 1, the research analysis indicated that 4 isolates were within 0 to 3 SNPs of each other and the fifth isolate was 32 SNPs different from the genetically closest isolate in the cluster. This was almost fully recapitulated by the NGD tool, with 4 isolates being within 0 to 3 SNPs of each other and the fifth isolate being 33 SNPs different from the genetically closest isolate in the cluster. The 3 isolates in cluster 2 were 31 to 43 SNPs different, with identical SNP differences being identified by the research pipeline and the NGD tool.

An epidemiological investigation was conducted for patients who were positive for the 4 highly related isolates in cluster 1 (0 to 3 SNPs different); this revealed that 2 patients were resident on the same ward (ward A) on overlapping admission dates and were known to the hospital infection control team. The third patient had an admission sample taken after transfer to a different ward but had overlapped with the first 2 cases on ward A prior to the ward transfer. The fourth patient was not an inpatient, and the sample had been taken in a hospital outpatient clinic that specialized in diabetic foot care. The infection control team had suspected an outbreak spanning ward A and a second ward (ward B) that was located on the same floor, on the opposite side of the corridor, but had not identified a link between the two. Ward A was a geriatric medicine ward, while ward B was commonly associated with the care of diabetic patients, and there were similar patient populations in these wards. The finding of a new patient in the outbreak, who had no history of admission to either ward A or ward B but had visited the diabetic foot clinic, indicated that the clinic could be playing a role in the outbreak. In response to the sequence data findings, infection control procedures, including health care worker screening, were implemented in the clinic. One health care worker was MRSA positive with an isolate that was 27 SNPs different from the 4 patient isolates, suggesting a more distant or indirect link.

## DISCUSSION

Here, we describe the findings of a preliminary evaluation of an automated bioinformatics system that enabled the real-time use of WGS data to determine pathogen relatedness, allowing the discovery of transmission and directing infection control interventions. The NGD tool demonstrated a high degree of accuracy, which is consistent with published studies on MRSA that were based on nonautomated informatics analyses (4, 12). The application of WGS coupled with automated relatedness determination enabled us to confirm an outbreak involving hospital inpatients and outpatients; this overcomes the bottleneck posed by the need for highly precise bioinformatics determination of relatedness, one of the final barriers to implementing MRSA sequencing as a critical component of infection control practices. The majority of isolates from our patients belonged to ST22, which is consistent with the known epidemiology of MRSA strains associated with carriage and disease in the United Kingdom (4). The NGD analysis tool was designed to be neutral regarding MRSA population structure, but more extensive evaluation with a larger and more genetically diverse isolate collection is now required.

The NGD tool also provided rapid automated prediction of phenotypic susceptibility. The current cost differential between phenotypic susceptibility testing and WGS precludes the use of sequencing as the principal testing methodology for MRSA. However, the generation of these data at no extra cost when sequencing for infection control reasons is performed provides a mechanism to build experience and data regarding the accuracy of genetic prediction in clinical practice, as well as an audit tool for the accuracy of routine laboratory phenotypic testing.

The analysis time for the 24 fastq files generated by a MiSeq run was 35 min (∼90 s per file/sample). There was no requirement for bioinformatics expertise to use the NGD system, although an understanding of the concept of bacterial genetic relatedness was required. This compares with up to 24 h to complete six steps using the standard research pipeline, which required an experienced bioinformatician. This time would increase as the number of isolates to compare increased.

Several alternative bioinformatics analysis tools are available for MRSA genomes, the majority of which are used by researchers and require considerable informatics expertise. Microreact is an open-access tool for the visualization and sharing of data on genomic epidemiology and phylogeography (13), and it has been used to describe a population snapshot of invasive S. aureus strains in Europe (11). Additional open-access research analysis platforms include Nullarbor (14) and a bacterial analysis pipeline provided by the Center for Genomic Epidemiology in Denmark (15). The recently introduced bioMérieux EpiSeq system (https://www.biomerieux-episeq.com) has been developed for clinical application and can analyze genomic data for 13 different bacterial species that are associated with hospital-acquired infections, including S. aureus. Data files (fastq or assembled fasta files) are uploaded and analyzed in a cloud service. Fee-for-service analyses include read assembly, bacterial identification to the species level, multilocus sequence typing (MLST) and *spa* typing, resistome and virulome characterization, and phylogenetic analysis. However, MLST- and *spa*-type-level relatedness is not adequate to ascertain transmission, and EpiSeq does not generate the SNP-level relatedness determinations required. EpiSeq was recently evaluated for investigating an increased incidence of S. aureus bloodstream infections in a neonatal intensive care unit in France (16). In that study, however, relatedness determination was performed with BioNumerics v7.6 (Applied Maths, Sint-Martens-Latem, Belgium), which is not automated, requires bioinformatics expertise to utilize, and has been reported to require overnight processing to determine the whole-genome multilocus ST (B. Magalhães, J. Goris, L. Senn, and D. S. Blanc, presented at the European Congress of Clinical Microbiology and Infectious Disease, Amsterdam, Netherlands, 13 to 16 April 2019).

In conclusion, the need for rapid, accurate, and automated tools that analyze bacterial WGS data and provide outputs that can be readily used without high-level bioinformatics expertise and interpreted by staff members in infection control and diagnostic microbiology units is widely acknowledged as an important barrier to the introduction of WGS into routine clinical laboratories. The growing developments in response to this obstacle indicate that effective solutions will become available in the near future.

## Supplementary Material

Supplemental file 1
